# Clinical outcomes and dosimetric study of hypofractionated Helical TomoTherapy in breast cancer patients

**DOI:** 10.1371/journal.pone.0211578

**Published:** 2019-01-31

**Authors:** Imjai Chitapanarux, Wannapha Nobnop, Damrongsak Tippanya, Patumrat Sripan, Somvilai Chakrabandhu, Pitchayaponne Klunklin, Wimrak Onchan, Bongkot Jia-Mahasap, Ekkasit Tharavichitkul

**Affiliations:** 1 Division of Radiation Oncology, Department of Radiology, Faculty of Medicine, Chiang Mai University, Chiang Mai, Thailand; 2 Northern Thai Research Group of Radiation Oncology (NTRG-RO), Faculty of Medicine, Chiang Mai University, Chiang Mai, Thailand; 3 Chiang Mai Cancer Registry, Maharaj Nakorn Chiang Mai Hospital, Faculty of Medicine, Chiang Mai University, Chiang Mai, Thailand; Roswell Park Cancer Institute, UNITED STATES

## Abstract

We present a single center’s experience of treatment outcomes and dosimetric parameters for breast cancer patients treated with hypofractionated Helical TomoTherapy (HT). This is a retrospective study of one hundred and thirty-six patients with invasive breast cancer treated between March 2012 and October 2016. Dosimetric parameters and 3-year loco-regional failure free survival (LRFFS) were analyzed. Dose to ipsilateral lung, heart and contralateral breast as well as acute and late toxicities were recorded. The median follow-up time is 45 months (range: 5–83). Two patients had loco-regional failure. The 3-year LRFFS was 99%. Acute grade 1 and 2 skin toxicities occurred in 95% and 1%, respectively. Coverage of the target volumes was achieved with the mean ± standard deviation (SD) of homogeneity and conformity index being 0.1 ± 0.04, and 0.8 ± 0.07, respectively. Dose to ipsilateral lung, contralateral breast, and heart was also within the limited constraints regardless of the complexity of target volumes. Only two percent of patients experienced late grade 2 skin toxicity. No late grade 2 subcutaneous tissue toxicity was found. Nine percent of patients developed late grade 1 lung toxicity. Hypofractionated radiotherapy using Helical TomoTherapy in breast irradiation provides excellent 3-year LRFFS and minimal acute and late toxicities. A careful, longer follow-up of healthy tissue effects to lung, heart, and contralateral breast is warranted.

## Introduction

A wide array of modalities is employed in the treatment of breast cancer, each with its own unique potential advantages and pitfalls. Options include surgical resection, radiotherapy (RT), and various combinations thereof. RT has demonstrated significant clinical benefits by decreasing the rate of recurrence and increasing the overall survival for patients who present a high risk of relapse after breast-conserving surgery or radical mastectomy [1]. But the benefits of RT in treating breast cancer are offset by the possible impact on cardiac toxicity and its dose non-uniformity [2–4]. This particularly applies to locally advanced tumors since RT partly covers organs at risk (OAR) such as the lungs or heart [5]. A more recent management trend, with limited but growing evidence for complex breast irradiation, is the use of Helical TomoTherapy (HT), which has shown to improve conformality to the tumor bed while sparing the OARs.

HT is a technique where multifield intensity-modulated radiotherapy (IMRT) is administered to a patient in motion along the rotation axis of a megavoltage X-ray source, offering unique 360-degree rotational irradiation [6]. This rotational-delivery approach around a single “virtual isocenter” may avoid the uncertainties inherent in multiple patient shifts. It presents dosimetric advantages compared to three dimensional conformal radiotherapy (3D-CRT) with regards to coverage of the target volumes [7], sparing the OAR with acceptable clinical tolerance, especially for patients with challenging anatomy or bilateral breast treatment. Furthermore, with HT there is no need to irradiate the nodal groups separately from the ipsilateral breast enabling the delivery of continuous craniocaudal irradiation along the entire extent of the disease, which reduces junction problems [8,9].

Since 2004, our center administers breast cancer irradiation with a hypofractionated schedule, both for whole breast and in the postmastectomy setting, with or without regional nodal irradiation (RNI), with 2D or 3D-CRT. This is done to improve patient convenience and provide better access to care. After installing the HT unit, Hi-ART II (Accuray, Incorporated, Sunnyvale, CA, USA) in March 2012 at our center, we started treating breast cancer patients with this approach. The following retrospective analysis describes early treatment outcomes and dosimetric parameters for breast cancer patients who were treated with hypofractionated HT at a single center.

## Materials and methods

### Patients

We retrospectively reviewed the medical records of patients who underwent breast or chest wall irradiation by HT between March 2012 and October 2016. Inclusion criteria were the following: patients with invasive ductal or lobular carcinoma, non-metastatic disease, and receiving adjuvant irradiation to breast or chest wall, no restrictions on regional nodal irradiation and prosthesis. We excluded patients with carcinoma in situ and pathology other than invasive ductal or lobular carcinoma. Recurrent cancer at chest wall or breast was also excluded from this study.

Twenty-four patients were excluded from the study due to the diagnosis of recurrent breast cancer, ductal carcinoma in situ, non-Hodgkin lymphoma of the breast, or phyllodes tumor. A total of 136 invasive breast cancer patients treated with HT with curative intent were evaluated. One-hundred and two patients (75%) received postmastectomy radiotherapy (PMRT) and 34 patients (25%) received postoperative radiotherapy after breast conserving surgery.

### Treatment planning and delivery

For postmastectomy patients, all patients were positioned on a wing board (CIVCO, Iowa, USA) with both arms raised above their head. Radiopaque wires were placed on the patients’ skin during computed tomography (CT) simulation to define the scars and field borders. Radiopaque wires were also placed around the palpable contralateral breast. The target volumes were treated with a dose of 265 cGy/fraction, 5 fractions per week to a total of 16 or 18 fractions in cases of a positive margin or T4d disease.

Images from CT-simulation were imported into the TomoTherapy treatment planning system, a planning station on Hi Art software, version 4.2.3 (Accuray, Incorporated, Sunnyvale, CA, USA). Chest wall, chest wall muscles, ribs, and pectoralis muscles were included in the chest wall planning target volume (CW PTV) for all stages except stage T1-2N0M0. CW PTV was restricted to a depth of 3 mm under the skin surface.

For breast conserving surgery patients, radiopaque wires were placed around the palpable breast tissue and over the surgical scar. Patients underwent CT scans with non-contrast from the lower neck to the upper abdomen at 5-mm-slice thickness. The target volumes were treated with a dose of 265 cGy/fraction with 5 fractions per week to a total of 16 fractions then sequentially boosted to the tumor bed with 200 cGy/fraction in 5–8 fractions. The breast clinical target volume (CTV) was delineated within the area outlined by the radiopaque wires around the ipsilateral breast. We followed the Radiation Therapy Oncology Group (RTOG) contouring atlas for breast cancer [10]. The breast planning treatment volume (PTV) was generated by expanding the margin by 5 mm around the breast CTV in all dimensions. For the tumor bed boost volume, a lumpectomy cavity was defined based on clinical information, surgical clips, and post-surgical changes such as seroma and fibrotic scars. The boost PTV was defined by expanding the margin by 5 mm around the boost CTV. Both boost and breast PTV were restricted to a depth of 3 mm under the skin surface to avoid hot spots on the skin surface and thereby reduce the potential for skin reactions and dose inhomogeneity. Typically, we retract PTV 1 voxel from the skin surface.

At our center, breast cancer patients with positive axillary lymph nodes are irradiated to breast or chest wall (CW) and regional nodes which includes supraclavicular (SPC) lymph nodes and/or level I-II axillary lymph nodes. Level III axillary lymph nodes are added in the case of clinical N2 disease at diagnosis or extracapsular invasion or inadequate axillary node dissection. Internal mammary lymph nodes are not included in our regional nodal irradiation (RNI) practice.

The organs at risk like ipsilateral lung, heart, and contralateral breast were delineated according to the RTOG Breast cancer atlas for radiation therapy planning: consensus definitions [10].

We used a HT plan in most cases, especially when we needed to irradiate the regional SPC lymph nodes. We used a TomoDirect plan in 8 cases, all of which were irradiated to the breast or chest wall only. We created a directional block to limit the entrance dose to OARs. The HT parameter definitions were 2.5 cm, 5 cm for the field width, 0.287, 0.215 for pitch, and 2.5–3.5 for modulation factor. The HT approach has been shown to provide sufficient photon fluence extending beyond the skin surface to allow up to 7-mm uncorrected setup error in the anterolateral direction [11]. For those plans created for the TomoDirect mode skin flash was applied to compensate for intra-fraction movement by retracting three leaves.

We tried to cover at least 95% of the PTV with a 95% isodose, to include a minimum PTV dose of 90% and a maximum dose of 107%. For the OARs, we attempted to limit the V25Gy for heart and V20Gy were minimized for ipsilateral lung. We tried to optimize the mean dose to the contralateral breast tissue to be less than 4 Gy [12]. Dose volume histograms (DVH) of the treatment plans were used in analyzing target coverage and doses to the organ at risks. The quality of the plans was assessed with the homogeneity index (HI) and conformity index (CI).

The HI was calculated according to the following formula: HI = (D2%–D98%)/D50%, where D2%, D98% and D50% were the minimum dose in 2%, 50% and 98% of the planning target volume, respectively. The CI of the combined PTV was calculated as follows: CI = (TVRI/TV) × (TVRI/VRI) (TV: Target Volume, TVRI: Target volume covered by the prescription isodose, VRI: volume of the prescription isodose) [13].

When the radiotherapy treatment started, the daily megavoltage CT (MVCT) images were acquired on the Helical TomoTherapy unit prior to each treatment fraction used for patient verification. Typically, the MVCT scan covers the entirety of the PTV.

### Outcome evaluation

Acute toxicities were assessed every week according to the RTOG/EORTC (European Organisation for Research and Treatment of Cancer) acute radiation morbidity scoring scheme [14]. Patients were evaluated for the treatment outcome and late radiation toxicities at 2–3 months interval for the first 2 years, and at 3–6 months interval between the third and fifth years. Late toxicities were assessed by the RTOG/EORTC late radiation morbidity scoring scheme.

Loco-regional failure free survival (LRFFS) was defined as the time that elapsed between treatment and local or regional recurrence/progression, or death due to breast cancer or unknown causes with undocumented site of failure. Overall survival (OS) was defined as the time from treatment to the date of death from any cause.

Both survival rates were estimated using the Kaplan-Meier method. *p*< 0.05 was considered statistically significant, and all p-values reported in this article are two-sided values, determined using Stata version 11 (StataCorp LP, College Station, TX, USA). This study was approved by the Research Ethics Committee of Faculty of Medicine, Chiang Mai University, Thailand.

## Results

### Patient characteristics

HT could be performed with the planned dose for all patients without any interruption. Patient demographics and treatment characteristics are shown in Table 1. Ninety-seven percent of patients received adjuvant chemotherapy before radiation therapy. Nearly half of our patients had left sided breast cancer.

**Table 1:** Patient and treatment characteristics.

Variable	Values (N = 136)
Age (Median; IQR) (year)	54 (47–60)
AJCC stage	
Stage I	7 (5.1%)
Stage II	52 (38.2%)
Stage III	71 (52.3%)
Stage IV	6 (4.4%)
Type of surgery	
BCS/MRM	34 (25%)/102 (75%)
Side	
Left/Right/Bilateral	67 (49.3%)/62 (45.6%)/7 (5.1%)
PTV	
Breast only	15 (11%)
Breast plus RNI	16 (11.8%)
CW plus RNI	88 (64.8%)
CW plus RNI plus silicone implantation	10 (7.3%)
Bilateral CW plus RNI	7 (5.1%)
RNI	121 (89%)
SPC only/SPC plus level III Axilla	25 (18.4%)/96 (70.6%)

**Abbreviations:** IQR, InterQuartile Range; BCS, breast conserving surgery; MRM, modified radical mastectomy; PTV, planning target volume; CW, chest wall; RNI, regional nodal irradiation; SPC, supraclavicular.

### Target volume and dosimetric parameters

Treatment volumes included breast only in 11%, breast plus RNI in 11.8%, chest wall plus RNI in 64.8%, chest wall with silicone implantation plus RNI in 7.3%, and bilateral chest wall plus RNI in 5.1%. The patient cohort in this study received RNI in 121 patients (89%); 25 of them received only SPC lymph node irradiation and 96 patients received level III axillary lymph node irradiation in the PTV due to clinical N2 disease at diagnosis in 17 patients, pathology of extracapsular invasion in 59 patients, and inadequate lymph node dissection in 20 patients. For all patients mean ± SD of HI and CI was 0.1± 0.04 and 0.8 ± 0.07, respectively. The mean ± SD of HI and CI in left and right sided breast cancer are mentioned in Table 2.

**Table 2:** Mean and standard deviations of the dosimetric parameters analysis.

Variables	Left side	Right side
PTV Breast only		
V90%	99.9±0.09	99.9±0.07
V95%	99.4±0.42	99.5±0.45
V107%	0.2±0.13	0.5±0.63
HI	0.1±0.01	0.1±0.02
CI	0.8±0.05	0.8±0.06
PTV Breast plus RNI		
V90%	99.9±0.09	99.9±0.12
V95%	99.2±0.70	99.3±0.56
V107%	0.6±0.80	0.2±0.36
HI	0.1±0.02	0.1±0.01
CI	0.7±0.06	0.8±0.09
PTV CW plus RNI		
V90%	99.9±0.19	99.9±0.06
V95%	98.8±0.49	98.7±0.56
V107%	0.3±0.29	0.4±0.76
HI	0.1±0.01	0.1±0.07
CI	0.8±0.05	0.8±0.06
PTV CW plus RNI plus silicone implantation		
V90%	99.9±0.05	99.8±0.16
V95%	99.7±0.24	98.5±1.11
V107%	0.1±0.10	0.9±1.04
HI	0.1±0.01	0.1±0.03
CI	0.7±0.10	0.8±0.09
PTV Bilateral CW plus RNI		
V90%	99.9±0.07	
V95%	98.8±0.44	
V107%	0.1±0.07	
HI	0.1±0.004	
CI	0.8±0.11	

**Abbreviations:** PTV, planning target volume; CW, chest wall, RNI; regional nodal irradiation; HI, homogeneity index; CI, conformity index

**Fig 1:**
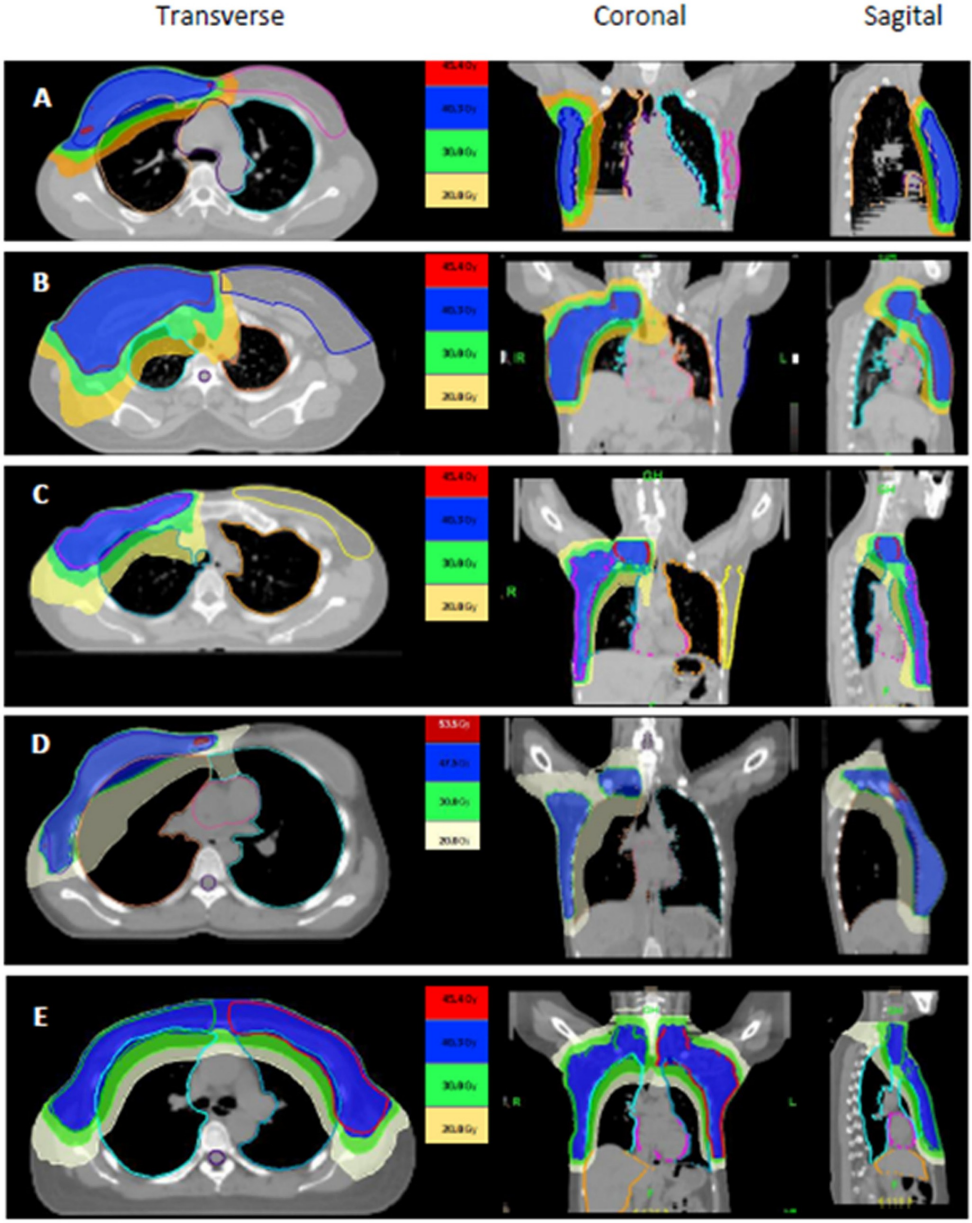
Dose distribution of transverse, coronal and sagittal plane by TomoTherapy treatment planning for (A) Breast only, (B) Breast plus regional nodal irradiation, (C) chest wall plus regional nodal irradiation, (D) chest wall plus regional nodal irradiation plus implantation and (E) Bilateral chest wall plus regional nodal irradiation.

For all patients, mean ± SD of V20Gy for ipsilateral lung was 33.6 ± 7.3%, mean ± SD of V25 Gy for heart was 9.7 ± 9.5% and mean ± SD of contralateral breast was 7.4 ± 2.1 Gy. Mean and standard deviations of the dosimetric parameters analysis for the organs at risks are displayed in Table 3, categorized by the different PTV and side of breast cancer. The examples of Helical TomoTherapy planning of different PTV are shown in Fig 1.

**Table 3:** Mean and standard deviation of the dosimetric parameters analysis for the organs at risks.

	Breast only		Breast plus RNI		CW plus RNI		CW plus RNI plus implantation		Bilateral CW plus RNI	
	Lt side	Rt side	Lt side	Rt side	Lt side	Rt side	Lt side	Rt side		
Ipsilateral lung									Lt lung	Rt lung
V20_Gy_ (%)	28.1±4.9	27.6±5.1	29.9±5.4	30.5±4.4	35.2±2.8	36.2±9.7	28.0±5.4	30.7±6.8	32.9±4.7	32.9±4.7
Mean dose (Gy)	16.4±2.8	15.6±1.4	17.1±2.0	17.0±1.3	18.4±1.1	19.0±1.9	17.1±1.3	17.6±1.6	18.0±1.9	18.1±2.3
Heart										
V25Gy (%)	14.6±8.3	2.2±1.7	17.2±5.2	3.4±2.9	12.4±8.7	5.2±6.5	11.7±6.6	8.1±5.5	24.0±16.0	
Mean dose (Gy)	17.1±3.5	11.0±3.1	17.5±2.1	12.6±1.8	15.5±3.3	12.2±2.9	16.1±2.9	14.7±2.3	18.7±4.5	
Mean contralateral breast (Gy)	6.5±1.2	7.8±2.9	7.4±1.9	7.8±2.7	7.4±1.8	7.3±2.1	8.4±2.8	7.2±2.7	-	

**Abbreviations:** PTV, planning target volume; CW, chest wall; RNI, regional nodal irradiation; Rt, right; Lt, left

#### Treatment outcomes

**Fig 2:**
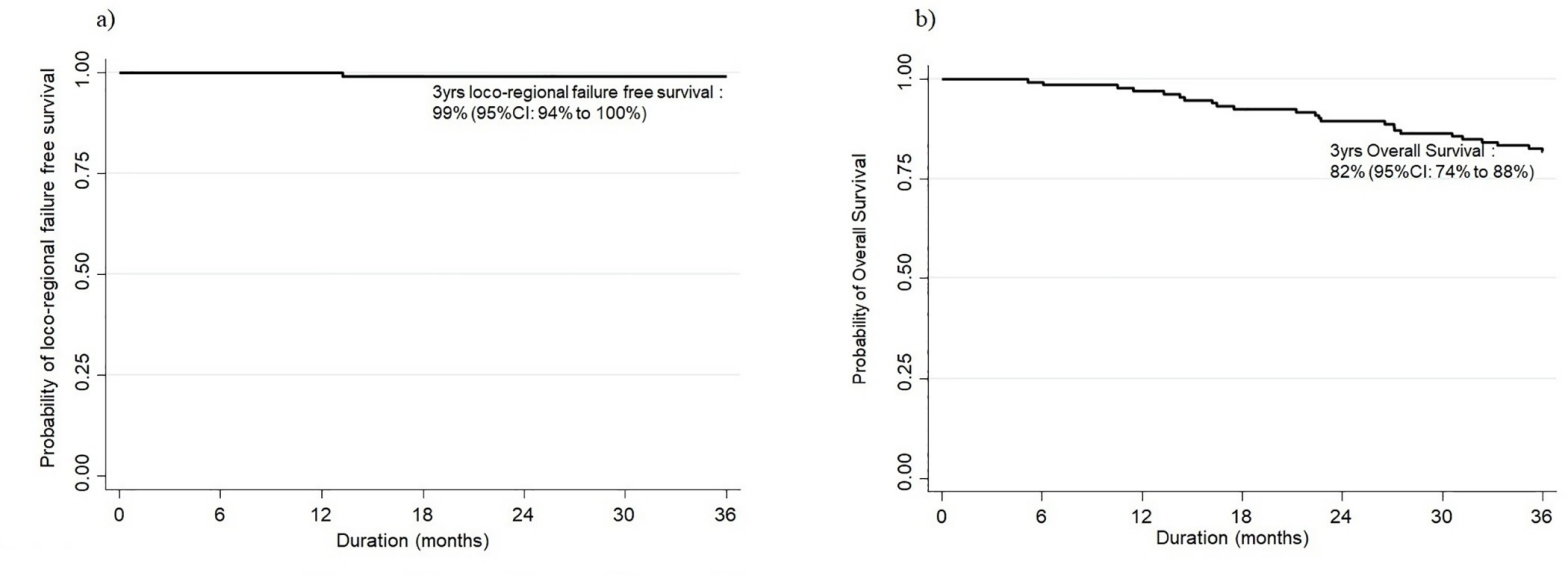
a) Kaplan-Meier estimate of 3-year loco-regional failure free survival. b) Kaplan-Meier estimate of 3-year overall survival.

At the time of analysis the median follow-up time was 45 months (range: 5 to 83), the 3-year loco-regional control rate was 99% (95% CI: 94% to 100%) and the 3-year OS was 82% (95% CI: 74% to 88%) (Fig 2A and 2B). Two patients had loco-regional failure with in-field recurrence.

### Acute and late toxicities

Grade 0, 1, and 2 acute skin toxicities were found in 4%, 95%, and 1%, respectively. For late toxicities we observed 1% grade 2 skin toxicities, 88% grade 1 skin toxicities, and 90% grade 1 subcutaneous tissue toxicity. Late lung toxicity could be assessed in 103 patients (76%) and only grade 1 was noted in 9%.

## Discussion

Over the years breast radiation oncology has focused on more personalized treatment planning taking individual patient’s anatomy into consideration. This has led to better disease control and reduced pulmonary and cardiac toxicity. HT is one such an approach for loco-regional breast radiation. Despite the increasing use of these loco-regional breast radiation techniques, there is a relative paucity of clinical studies in Southeast Asia. The present study demonstrates how HT allows breast cancer radiotherapy to adapt to a patient’s anatomy, deliver optimal coverage of treatment volumes, and minimize dose to OAR. Thus, our study provides further support for the feasibility of this technique from the Thai standpoint. Best outcomes can be achieved by individualizing the approach and combining HT with correct patient positioning, treatment planning, and delivery.

HT improves target coverage while sparing OAR because of its ability to achieve a higher degree of conformity to the PTV. The superiority of HT in terms of PTV coverage, normal tissue sparing and dose homogeneity has been demonstrated in previous studies [15–16]. Camarota et al. [17] conducted a dosimetric study on 12 breast cancer patients comparing two different hypofractionated radiation techniques: 3D-CRT in prone position and HT in supine position. They demonstrated that PTV coverage was achieved with both techniques but HT plans offered superior dose homogeneity. Haciislamoglu et al. [18] evaluated the dose distribution and homogeneity of four different types of Intensity Modulated Radiation Therapy (IMRT) (forward-planned IMRT, inverse-planned IMRT, HT and VMAT) in comparison with standard wedged tangential-beam 3D-CRT of the left breast in post-lumpectomy patients. They concluded that among the evaluated modalities, HT resulted in the lowest maximum dose to the ipsilateral organs, hence concluded to be better than the other options. In our study, dose coverage of the PTV was higher than 98% of V95 for all groups and the PTV breast only showed the highest target coverage of 99.9 ± 0.09 for the left side and 99.9 ± 0.07 for the right side. But target coverage tends to decrease in the PTV with RNI. Regarding the OAR’s dosimetric assessment, the breast only PTV showed the lowest mean dose to the ipsilateral lung, heart and contralateral breast. The mean dose was higher for the PTV breast plus RNI and chest wall plus RNI. The PTV CW+RNI+ silicone implantation demonstrated a lower mean dose of the ipsilateral lung when compared to the PTV CW+RNI, whereas the mean dose of the heart was higher. For PTV bilateral CW plus RNI, the mean dose and V20Gy for ipsilateral lung was found to be lower than the PTV CW plus RNI. However, PTV bilateral CW plus RNI showed the highest mean dose and V25Gy for the heart.

The CI and HI were not significantly different between all groups of PTV with the average value of 0.8 ± 0.07 for CI and 0.10 ± 0.04 for HI. Like our study, Qiu et al. [19] demonstrated a CI of 0.8 for whole breast radiotherapy with HT. Zhou et al [16] reported HI and CI to be 1.1 ± 0.03 and 0.7 ± 0.12 with HT respectively. They further concluded that compared to SaS-IMRT (Step and Shoot IMRT) and 3D-CRT, HT technique in treating breast cancer had the best CI and HI as well as the steepest dose gradient due to its highly modulated beamlets with rotational technique.

Comparing between the left and right sided target, there was no significant difference for target coverage of V95, HI and CI and their p-values were 0.384, 0.128 and 0.215, respectively. Moreover, the mean organ doses for ipsilateral lung and contralateral breast were not significantly different between the left and right sided target with p-values of 0.887 and 0.860, respectively. However, the right-sided target had a significantly lower mean dose to the heart with *p* = 0.031 and V25Gy with *p* = 0.029. On average the mean dose was 12.6 Gy (right) and 16.6 Gy (left) and V25Gy was 4.7 Gy (right) and 14.0 Gy (left). Goddu et al. [5] generated the HT treatment plan for left-sided advanced breast cancer. Their plan described a lower dose for ipsilateral lung and heart than our plan in all situations. Comparing our treatment plan with Goddu et al, the mean percentage of the ipsilateral lung volume receiving 20 Gy or more in their plan was 17.6 ± 3.5%, while in our plan it was 29.9 ± 5.4%, 35.2% ± 2.8%, and 28.0% ± 5.4% for left breast plus RNI, left chest wall plus RNI, and left chest wall plus RNI plus implantation, respectively. Their mean heart dose was 12.2 ± 1.8 Gy whereas our results show 17.5 ± 2.1, 15.5 ± 3.3, and 16.1 ± 2.9 for left breast plus RNI, left chest wall plus RNI, and left chest wall plus RNI plus implantation, respectively. The explanation for the heart dose would be (1) variability in heart contouring among radiation oncologists (RO) (2) left sided breast cancer in 49.3% of our study population (3) the chest wall PTV overlapped with the heart volume in some cases. The patients in this cohort were contoured by 6 ROs at our center. Some ROs included the left anterior descending coronary artery (LAD), whereas the others contoured the heart only. Taylor et al. [4] have suggested that the maximum heart distance in a treatment field, measured anterior to posterior, correlates with mean heart dose.

Our loco-regional outcome was in accordance with the published literature. Chira et al. [20] used neoadjuvant radio-chemotherapy with HT for 5 patients with locally advanced breast cancer. All patients underwent mastectomy after radiotherapy. The preliminary results of Chira et al. showed that HT is feasible for neoadjuvant radiotherapy with acceptable toxicity profiles. Nagai et al. [21] reported the 3-year local control rate, regional control rate, and overall survival of 99.1%, 98.6%, and 97.7% in breast cancer patients treated by two-beam TomoDirect IMRT. Similarly, the retrospective study by Duma et al. [22] reported their experience of using HT to treat 26 very large and irregular shaped loco-regional advanced breast cancer and recurrent breast cancer lesions. They reported the 2-year LRFS of 84%. The median LRFS and OS after radiotherapy was 21 and 57 months for the primary group versus 10 and 11 months for the recurrence group.

One of the current challenges for radiation oncologists treating breast cancer patients is that the multiple adjacent fields lead to hot and cold spots at the field junctions when treating the lymph nodes surrounding the breast. In our cohort, 121 patients received RNI, 25 of them received only SPC lymph node irradiation and 96 patients received level III axillary lymph node irradiation in the PTV due to clinical N2 disease at diagnosis in 17 patients, pathology of extracapsular invasion in 59 patients, and inadequate lymph node dissection in 20 patients. HT has not only the ability to correct setup errors but also the capacity to deliver a continuous craniocaudal dose, which eliminates field junctions.

Currently, another aim of whole breast radiotherapy is the reduction of acute and late patient toxicity. In our study, with the dose constraints used for PTV and normal tissue, the acute toxicities observed were low. Grade 0, 1, and 2 acute skin toxicity was found in 4%, 95%, and 1%, respectively. Duma et al [22] reported Grade 0, 1, and 2 skin toxicity to be 57%, 14%, and 28% respectively and Candales et al. [23] reported grade 1 and 2 acute skin toxicity in 41.7% and 58.3% respectively. The reason for higher toxicity reported by other studies could be due to complex treatment volumes and higher total prescribed dose compared to our study. For late toxicities we found only 1% of grade 2 skin toxicities, 88% of grade 1 skin toxicities, 90% of grade 1 subcutaneous tissue toxicity, and 9% of grade 1 lung toxicity (only 103 patients available for assessment). The report on late toxicity from HT is scarce, however, Duma et al [22] reported late lung toxicity in 1 of 6 patients who had longer follow-up available.

In conclusion, hypofractionated radiotherapy by HT in breast irradiation allows a satisfactory dose conformity and homogeneity index with an acceptable acute tolerance and should be considered a viable option in complex adjuvant breast and nodal irradiation. Also, it provides an excellent 3-year loco-regional failure free survival and minimal acute and late skin and subcutaneous toxicities. A careful, longer follow-up of healthy tissues like lung, heart, contralateral breast is warranted.

## Supporting information

S1 Data Data used in this study. (XLS)
